# The Effect of Breathing Exercises on the Nocturnal Enuresis in the Children with the Sleep-Disordered Breathing

**DOI:** 10.5812/ircmj.8986

**Published:** 2013-11-05

**Authors:** Shahnaz Khaleghipour, Mohsen Masjedi, Roya Kelishadi

**Affiliations:** 1 Department of Psychology, Naein Branch, Islamic Azad University, Naein, IR Iran; 2 Department of Immunology, Isfahan University of Medical Sciences, Isfahan, IR Iran; 3 Child Growth and Development Research Center, Isfahan University of Medical Sciences, Isfahan, IR Iran

**Keywords:** Breathing Exercises, Nocturnal Enuresis, Child

## Abstract

**Background:**

The nocturnal enuresis is one of the most common complaints of childhood. Upper airway obstruction and nocturnal snoring affect the nocturnal enuresis in children.

**Objectives:**

The aim of this study was to investigate the effects of breathing exercises on the nocturnal enuresis in the children with the sleep-disordered breathing.

**Patients and Methods:**

This study was conducted in year of 2011 by a semi-experimental design with the control group among 40 children, aged 6 - 12 years, who had the nocturnal enuresis. Participants were examined based on the criteria of nocturnal enuresis, oral breathing, and nocturnal snoring. Subsequently, they were randomly assigned to the case and control groups. In the case group, the breathing exercises were performed for 45 minutes, and were pursued for four weeks in the morning following and prior to sleeping, and subsequently the arterial blood gases were measured and the frequency of enuresis and the respiratory rates (RR) were recorded.

**Results:**

After intervention the means of PaCO_2_ and RR in the control group were significantly higher than the case group (P < 0.0001). Likewise, O_2_sat, PaO_2_ in the case group were higher than the control group (P < 0.0001). The nocturnal enuresis decreased significantly in the case group, compared to the control group (P < 0.0001).

**Conclusions:**

This study suggests that the breathing exercises may reduce the frequency of nocturnal enuresis in the patients with the oral breathing and nocturnal snore. The clinical implications of these findings should be verified in the future longitudinal studies.

## 1. Background

Nocturnal enuresis is defined an accidental urination with at least, twice per week for three consecutive months, and calendar age of five years old (1). It is one of the most common complaints of childhood; children from the age of five should be able to control the urine (2, 3) because at this age, bladder capacity increases and brain nerve centers control the urinary bladder contractions during sleep (4). Many children cannot reach this stage. Therefore, children and their families face severe behavioral and social problems (1). Various methods have been proposed for the treatment of nocturnal enuresis, none of which is of a definitive efficacy (2, 3, 5). One of the findings concerning this disorder is that the nocturnal enuresis in children with the sleep-disordered breathing, whose nostrils are blocked, happens more (6-8). Relaxed muscles which help to open the airways and soft palate vibrations during sleep, will cause snoring, while in the normal condition air enters through the nose during the aspiration and slip on the edge of the soft palate. If this little edge moves and makes a barrier to the air, the sound of snoring is created (8, 9). Chang et al. (10) and Yeung et al. (11) have shown that the prevalence of nocturnal enuresis in children with the habitual snoring is four times more and 46 percent of children with the sleep- disordered breathing syndrome have the nocturnal enuresis (12). In some children, recurrent upper respiratory tract persistent infections, adenoids, habitual oral breathing, blocked Eustachian tube and nocturnal snoring can cause enuresis (13, 14). Children's physical health is dependent on how they are breathing. The natural activity of respiratory tract is controlled through the neck muscles, inter-rib muscles, abdominal muscles, and diaphragm. Some of the breathing disorders occur when the transferred messages through the brain are unable to transfer to these muscles through the spinal cord (15, 16). In children with the oral breathing, the superficial and shallow breathing create the nocturnal snore due to increasing weakness of breathing muscles. This factor causes the bladder to lose its control on urination (17, 18). Ezzat waleeda et al. have shown that opening blocked airway by the surgery will reduce the nocturnal enuresis in the children with the respiratory disorders (16). Breath control is done by the aspiration and expiration. In breathing exercises, the individual capacity will increase and the equilibrium is established between the nervous and respiratory systems and the mind will work normally (19), so that by the deep breathing, the peripheral air amount will also increase substantially. Respiratory exercises, tolerance exercises and exercise activities related to the upper and lower parts improve breathing process and create a balanced ventilation system (20). The reason is that individuals under the influence of respiratory exercises show an increase in the lung functional indices, and thus, their oxygen consumption volume rises (18, 21). A few external studies are present concerning the effect of breathing exercises improvement on the children nocturnal enuresis with the conflicting results, but we were unable to find any internal reference in this regard (15, 17, 22).

## 2. Objectives

Therefore, the aim of this study was to investigate the effects of breathing exercises on the reduction of the nocturnal enuresis in the children with the sleep-disordered breathing.

## 3. Patients and Methods

### 3.1. Patients

This study was conducted in year of 2011 by a semi-experimental study with a control group. The control and case groups were selected based on the DSM-IV-TR criteria by the convenience sampling (23). The groups were children, who had the nocturnal enuresis, and hence; were brought to the kidney and urethra clinic affiliated to the Isfahan University of Medical Sciences by their parents. The children’s problem of breathing was also diagnosed by the ear, nose and throat specialists. Both groups were consisted of 40 children (6-12 years old) with the concomitant nocturnal enuresis and oral breathing. The 40 children were classified into the two equal groups. Participants were examined based on the criteria of oral breathing, nocturnal snoring, and nocturnal enuresis two times per week and the sleep-disordered breathing confirmed by the otolaryngologists. The exclusion criteria consisted of the current drug consumption (as, tetracycline, nitrofurantoin, methicillin, acethazolamide may decrease the level of PaCO_2_, whereas bicarbonate, hydrocortisone, metolazone may increase the level of PaO_2_), (24) mental and behavioral disorders, tonsillitis, physical disability, mental retardation, allergies and cigarette smoking by the parents.

### 3.2. Procedure

First nocturnal enuresis frequency and the RR were recorded in the case and control groups. The RR was measured when the children were at rest and involved counting the number of breaths for one minute by counting how many times the chest rose. In addition, the patients of both groups were analyzed for the blood arterial gases. Subsequently, breathing exercises were taught to the children and their parents in the case group by the demo games and video clips by speech therapist. The patients did not drink water/any other liquids two hours before sleep. Then breathing exercises pursued in four weeks in the morning after getting-up, and also at night before sleep. Firstly, the breathing exercises of hip, anal and neck muscles and diaphragm were performed. For the evacuation of abdominal breathing toward the spine, diaphragm was drawn to the ribs. Then, the movements of the head and spinal column spin the effective energy in the body. During these exercises, breathing was done by the right nostril. Then, breathing exercises of the nose and jaw were done with closing the right nostril and pushing the chin to the chest, while expiration was done by the left nostril. Finally, during abdominal exercises, the inspiration was retained and with mouth closing, expiration expelled through the right nostril (14, 23). Then, the frequency of nocturnal enuresis and the RR were recorded. Upon the completion of breathing exercises, the arterial blood gases were also measured.

### 3.3. Collection and Handling of Blood Samples

A radial arterial blood of five milliliters was drawn fasting by the air-free bubbles plastic syringes (gauge 23) containing lyophilized heparin. Subsequently, the blood samples under the condition of air-sealed were placed on the ice bag instantly, and sent in a pre-cold chamber (approximate temperature 4 ˚C) to the laboratory within 10 minutes for the blood gases analysis. The laboratory also carried out the assay within 30 minutes (24). The arterial blood gas analyzer (ABL 300, Radiometer, Copenhagen, Denmark) was employed to measure the oxygen, carbon dioxide and pH levels.

### 3.4. Statistical Analysis

 Statistical analyses were performed using SPSS software, version 16.0. Data presented as mean ± SD, median [IQR] and number as appropriate. At the baseline age, weight, height, body mass index (BMI), O_2_sat, PaO_2_, PaCO_2_, pH and RR were compared before and after the study in the control and case groups, using the independent, paired t-tests, and repeated measurements of ANOVA. In addition, the Chi-Square test was used to assess the distribution of sex between the groups. Likewise, nocturnal enuresis frequency was compared, before and after the study within and between the groups, using the Wilcoxon Signed Rank and Mann-Whitney tests, respectively. Statistical significance was accepted at the p-value less than 0.05.

### 3.5. Ethical Consideration

This study was approved by the Ethics Committee of the Isfahan University of Medical Sciences. Written informed consent was obtained from the parents, and that the study protocol conformed to the ethical guidelines of the 1975 Declaration of Helsinki as reflected in a priory approval by the institution's human research committee.

## 4. Results

Table 1 displays the total number of the case group consisted of three girls and 17 boys. The case group was between 6 to 10 years old, with a mean age of 7.09 ± 1.23. In addition, the control group was between 6 to 11 years old, with a mean age of 8.12 ± 1.74. The results showed that there was insignificant difference in gender between the two groups. In addition, there was insignificant difference in the BMI, weight, and height between the case and the control groups. 

**Table 1.** Demographic Characteristics of the Groups.

	Case group ^a^	Control group ^a^	P value
**Weight, kg**	29.5 (2.81)	29.3 (2.16)	0.824 ^b^
**Height, cm**	127.4 (2.19)	126.9 (2.04)	0.945 ^b^
**BMI, kg/m2**	15.92 (2.31)	16.04 (1.08)	0.129 ^b^
**Age**			
6-11	7.09 (1.23)	8.12 (1.74)	0.421 ^c^
**Gender**			
Male	3	6	
Female	17	14	0.189 ^b^

^a^ Values are represented as mean (SD)

^b^ P values were calculated by the Chi Squared

^c^ P values were calculated by the independent t-test

Table 2 displays the comparison of the means of the O _2 _sat, PaO _2 _, PaCO _2 _, pH and RR between the groups before and after intervention. Before intervention, the means of O _2 _sat, PaO _2 _, PaCO _2 _, pH and RR were similar in both groups (P > 0.05). After intervention, only pH was similar in both groups (P > 0.05) and the means of O _2 _sat, PaO _2 _in the case group were significantly higher than the control group (P < 0.0001). However, in the control group, the means of the PaCO _2 _and RR were significantly higher than the case group (P < 0.0001). 

**Table 2.** Comparison of the O_2_sat, PaO_2_, PaCO_2_, pH and RR between the Groups.

	Control Group ^a^		Case Group ^a^		P value ^b^
	Before	After	Before	After	
**O_2_sat, mm/Hg ^c^**	84.61 (1.13)	84.26 (1.21)	83.92 (3.29)	89.45 (2.17)	0.001
**P value ** ^**d**^	0.92		0.01		
**PaO, mm/Hg_2_, mm/Hg^e^**	69.54 (1.26)	67.20 (2.14)		75.28 (2.43)	0.001
**P value ** ^**d**^	0.091		0.001		
**PaCO_2 _, mm/Hg **	75.16 (1.56)	76.12 (1.23)	72.30 (1.49)	60.21 (1.28)	0.001
**P value ** ^**d**^	0.34		0.001		
**pH, mm/Hg**	7.76 (1.34)	7.43 (1.23)	7.91 (2.28)	7.30 (2.19)	0.124
**P value ** ^**d**^	0.87		0.12		
**RR ** ^**f**^	29.61 (1.21)	29.12 (1.61)	27.42 (1.06)	21.34 (1.16)	0.001
**P value ** ^**d**^	0.91		0.01		

^a^ Values are represented as mean (SD)

^b^ P-values were calculated by the repeated measurements of ANOVA

^c^ P-values were calculated by the Oxygen 2 saturation

^d^ P-values were calculated by the paired t-test.

^e^ P-values were calculated by the partial pressure of O_2_

^f^ P-values were calculated by the Respiratory Rates

**Figure 1.**
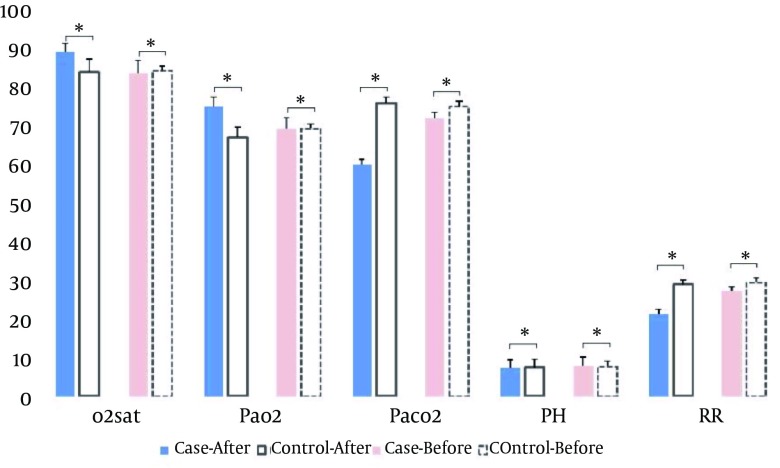
Depicts the Comparison of the Means of the O _2 _sat, PaO _2 _, PaCO _2 _, pH and RR Between the Groups Before and After Intervention. Before intervention, there were insignificant alteration in the means of O_2_sat, PaO_2_, paCO_2_, pH and RR between both groups (P > 0.05). However, after intervention, the means of O_2_sat, PaO_2_ in the case group were significantly higher than the control group (P < 0.0001). Conversely, the means of the PaCO_2_ and RR in the control group were significantly higher than the case group (P < 0.0001).

Table 3 displays the comparison of the median of nocturnal enuresis frequency between the groups before and after intervention. Before intervention, the median of nocturnal enuresis frequency was similar in both groups (P > 0.001). After intervention, the median of nocturnal enuresis frequency in the case group was significantly lower than the control group (P < 0.001). 

**Table 3.** Comparison of the Nocturnal Enuresis Frequency between the Groups.

	Before	After	P value ^a^
**Case Group ^b^**	6 (4.25 – 8)	1 (0 – 2)	< 0.0001
**Control Group ** ^**b**^	6 (4.25 – 7.75)	5.5 (4 – 8.75)	0.83
**P value ** ^**c**^	0.7	< 0.0001	

^a^ P-values were calculated by the Wilcoxon Signed Rank tests,

^b^ Values are represented as median (IQR).

^c^ P-values were calculated by the Mann-Whitney.

## 5. Discussion

This study aimed to consider the effects of breathing exercises on the nocturnal enuresis in the children with the sleep-disordered breathing. Normally, in each breath, 500 ml of air enter the lungs, and then it goes out of the lungs after the exchange of oxygen and carbon dioxide. With deep breathing, the amount of air can be increased largely. These breathing exercises bring the air volume to 2500 ml in each breath. Therefore, by breathing exercises, more air will enter the lungs, arterial blood oxygen saturation will increase, and respiratory proper functioning will be possible, which promotes deep breathing improvement (11, 25, 26). Breathing exercises will also prevent the accumulation of secretions and atelectasis, strengthen the respiratory muscles, coordinate aspiration muscles and correct the breathing patterns. It also decreases the asthmatic attacks and eliminates the dyspnea (14). On the contrary, in children with the oral breathing during sleep, the upper airways endure vibration due to airflow, and breathing becomes difficult due to weakening of the respiratory muscles. In addition, the chronic superficial breathing causes the weakening of the inter-rib muscles and diaphragm muscle (17, 18). Many studies show that the removal of airways blockade even in case of surgery, reduces the frequency of nocturnal enuresis in children with the breathing disorders (16). There are some drugs and various behavioral to treat the nocturnal enuresis, but none of them is crucial, and each has side effects. On the other hand, the treatment of nocturnal enuresis with respect to the associated disorders, can be more effective/with less complications (2, 27, 28).

The results of this study showed that in children with the nocturnal enuresis, who had the blockade of airways/abnormal breathing pattern; the breathing exercises can be used as a simple method with low expenses and more efficient than other approaches. The findings of this study also showed that there was no significant difference in the demographic characteristics between the two groups (Table 1). In addition, the findings of this study revealed that there was a significant difference in the mean frequency of nocturnal enuresis between the case and the control groups after the intervention (Table 3). These findings demonstrate that the frequency of breathing exercises reduce the nocturnal enuresis in the children with the sleep-disordered breathing. There are many studies concerning the relationship between the nocturnal enuresis and nocturnal snore/airways blockade ( 16 , 21 , 22 , 29 ). The nocturnal enuresis is more common in children with the oral breathing. It appears that the relative hypoxia in these children causes low-pressure oxygen in the distal tubes of kidney, unresponsiveness to the anti-diuretic hormone, and increased production of urine temporarily, particularly during the sleep. Both of these factors can increase the volume of urine, and consequently make the urine control more difficult and finally lead to the nocturnal enuresis ( 3 , 7 ). As far as we are concerned, there is no internal research concerning the impact of breathing exercises on the nocturnal enuresis in children. Nevertheless, Crouch et al. have shown that in subjects with the breathing disorders, the pulmonary rehabilitation programs including breathing exercises, endurance activities, upper and lower extremities exercises like purse lip breathing and diaphragmatic breathing, improve ventilation and in turn, have caused the adequate exchange of air pattern ( 19 ). Ghahri Sarabi et al. have also shown that in patients with the breathing disorders, breathing exercises increase the lung functional indices ( 27 ). In addition, another study has shown that in patients with the respiratory chronic diseases, pulmonary rehabilitation and breathing exercises increase the volume of oxygen consumption ( 20 ). The respiratory disorders cause ineffective breathing habits and shallow breathing. This chronic shallow breathing leads to the weakening of the diaphragm muscle and inter-costal muscles ( 8 , 19 ). In conclusion; these findings suggest that the breathing exercises may reduce the nocturnal enuresis in the children with the sleep-disordered breathing. The clinical implications of these findings should be verified in the future longitudinal studies. 
